# Genotyping data of French wild boar populations using porcine genome-wide genotyping array

**DOI:** 10.1186/s13104-022-06052-w

**Published:** 2022-05-13

**Authors:** Nathalie Iannuccelli, Nicolas Mary, Nathalie Bonnet, Geoffrey Petit, Carine Valle, Alain Ducos, Juliette Riquet

**Affiliations:** 1 GenPhySE, Université de Toulouse, INRAE, ENVT, 31326 Castanet-Tolosan, France; 2 ASTRE, CIRAD, INRAE, 34398 Montpellier, France; 3 Technological Center, Genomics and Transcriptomics Platform, Cancer Research Center of Toulouse, Toulouse, France; 4 INSERM, UMR 1037, Toulouse, France

**Keywords:** Wild boar, Genome-wide DNA genotyping, SNP, Porcine microarray

## Abstract

**Objective:**

The admixture of domestic pig into wild boar populations is controlled until now, by cytogenetic analysis. Even if a first-generation hybrid animal is discernable because of its 37-chromosome karyotype, the cytogenetic method is not applicable in the case of advanced intercrosses. The aim of this study is therefore to evaluate the use of SNP (Single Nucleotide Polymorphism) markers as an alternative technology to characterize recent or past hybridization between the two sub-species. The final goal would be to develop a molecular diagnostic tool.

**Data description:**

The Geneseek Genomic Profiler High-Density porcine beadchip (GGP70KHD, Illumina, USA), comprising 68,516 porcine SNPs, was used on a set of 362 wild boars with diverse chromosomal statuses collected from different areas and breeding environments in France. We generated approximately 62,192–64,046 genotypes per wild boar. The present dataset might be useful for the community (i) for developing molecular tools to evaluate the admixture of domestic pig into wild boar populations, and (ii) for genetic diversity studies including wild boar species or phylogeny analyses of Suidae populations.

Raw data files and a processed matrix data file were deposited in the ArrayExpress at European Molecular Biology Laboratory-European Bioinformatics Institute (EMBL-EBI) data portal under accession number E-MTAB-10591.

## Objective

Different studies have reported that wild boar populations have significantly increased in France, like in other parts of the world [1]. Among the different hypothesis, the mating of domestic pigs with wild boars can partly explain this expansion [2]. The growth of the wild boar population today is associated with many problems such as crop damage, increased health risks for livestock and decreased biodiversity. Consequently, the genetic status of the wild boar populations has been monitored by cytogenetic analysis since the 1980s in France [3]. Indeed, the karyotype of the wild boar (2n = 36) differs from that of the domestic pig (2n = 38) due to a Robertsonian translocation between chromosomes 15 and 17. However, this method is not relevant enough to guarantee the “wild boar status” of an animal with 36 chromosomes resulting, for example, from the mating of two parents with 37 chromosomes. Therefore, the aim of this analysis was to evaluate the use of molecular markers as an alternative technology. A genome-wide high-throughput genotyping approach had not yet been performed on a large number of French wild boars; only a small-scale molecular approach has recently been evaluated [4, 5]. In addition, chromosomal control requires metaphase spreads prepared from cultures of peripheral blood lymphocytes obtained in fresh whole heparin blood samples, which can be very difficult to collect on wild animals. The final aim would be to develop a robust and practical molecular diagnostic tool to replace cytogenetic analyses.

The data set is still confidential but a scientific publication is in progress and initial analyses have already been presented in an oral communication [6]. In the future, the use of molecular markers for wild boar genotyping should make it possible to refine our knowledge about the consequences of hybridization phenomena on the phenotypic traits of wild animals and to evaluate if the evolutions observed in wild populations can result from the genetic introgression of alleles from the domestic pig.

The present data set might be useful for genetic diversity studies to improve wild species preservation measures in our country. In addition, these all-genome genotyping data from the French wild boar populations could be useful for phylogeny studies of Suidae populations.

## Data description

### Animals

The experiment included 362 French wild boars from different French regions and environments. Most of the animals (n = 252) were bred in enclosed wild boar farms spread out over 38 French administrative subdivisions. The other animals were bred in free-range conditions, either in a protected area (French administrative subdivision, “Deux-Sèvres”; n = 31), or in an unprotected area (French administrative subdivision, “Ardèche”; n = 79). There were 223 females and 139 males. According to the cytogenetic analysis, there were 203 animals with chromosome numbers equal to 2n = 36, 70 animals with 2n = 37, 10 animals with 2n = 38, and 79 animals with unknown chromosomal status. No animals in our study were bred/killed/taken specifically for the needs of our project, which therefore did not require explicit authorization (in accordance with the 2010/63/EU European Directive).

### Sample collection

A total of 362 biological samples were collected. The biological samples of animals from free ranging “Ardèche” were ear biopsies (n = 79) collected between the years 2014–2016 for an edema disease project [7]. The other samples were blood samples (n = 283) collected between the years 2017–2019, analyzed on the chromosomal control platform [3] and selected to be representative of the farm diversity.

### DNA extraction

DNA samples were extracted for the 362 biological samples. Genomic DNA of 283 heparin blood samples were extracted using the Blood DNA Isolation (Norgen) kit. Genomic DNA of 79 ear biopsies were extracted with an in-house protocol (proteinase K lysis followed by salt-based DNA extraction and ethanol precipitation). Total genomic DNA quality was determined using the Nanodrop 8000 spectrophotometer (ND8000LAPTOP, Thermo Fisher Scientific, USA). Total genomic DNA concentration was determined using the Quant-iT picogreen dsDNA Assay Broad Range kit (Invitrogen, Q33130, Thermo Fisher Scientific, USA) with the QuantStudio6 instrument at the Genomic and Transcriptomic (GeT) platform [8]. All the samples were managed with the GeT Barcode database.

### DNA genotyping experiment and raw data

A total of 362 genomic DNA samples were genotyped in collaboration with the Cancer Research Center of Toulouse (CRCT) core facility platform [9]. Genotypes were performed using the GGP70KHD porcine array comprising 68,516 porcine SNPs. The Infinium High Density Ultra Assay protocol (Illumina, USA) was performed according to the manufacturer’s recommendations. The whole data set was produced on 16 beadchips. Raw data were processed with an Array Scanner iScan System (Illumina, USA) instrument, and 724 raw data files (Table 1 Data set 1 and Data set 2) were deposited in ArrayExpress at the EMBL-EBI data portal under accession number E-MTAB-10591 [10].

**Table 1:** Overview of data set

Label	Name of data file/data set	File types (file extension)	Data repository and identifier (DOI or accession number)
Data set 1	E-MTAB-10591.raw.1	Raw data files (.zip)	Array express, https://identifiers.org/arrayexpress:E-MTAB-10591, [10]
Data set 2	E-MTAB-10591.raw.2	Raw data files (.zip)	Array express, https://identifiers.org/arrayexpress:E-MTAB-10591, [10]
Data set 3	E-MTAB-10591.processed.1	Processed matrix data (.zip)	Array express, https://identifiers.org/arrayexpress:E-MTAB-10591, [10]

### DNA genotyping analysis

Genotypes were inferred from the raw fluorescence intensity data using the Genotyping Analysis Module included in GenomeStudio (version 2.0.5) software (Illumina, USA). We used a custom cluster from the commercial cluster file and Illumina guidelines [11]. The call rate of wild boar samples is approximately 0.91–0.93, and we generated approximately 62,192–64,046 genotypes per DNA sample. The genotyping report of all samples was exported in a single file with top format alleles, including B Allele Freq and Log R Ratio for potential CNV (Copy Number Variation) analysis. The processed matrix data file (Table 1 Data set 3) was deposited in ArrayExpress at the EMBL-EBI data portal under accession number E-MTAB-10591 [10].

## Limitations

The call rate of wild boar samples is approximately 0.91–0.93. Quality of wild boar genotyping using porcine array depends on the quality of heterologous hybridization.

## Data Availability

The data presented in this Data Note can be freely and openly accessed on the EMBL-EBI ArrayExpress portal under accession number E-MTAB-10591, https://identifiers.org/arrayexpress:E-MTAB-10591 [10].

## Abbreviations

SNP: Single nucleotide polymorphism
GGP70KHD: Geneseek genomic profiler 70 K high density
EMBL-EBI: European molecular biology laboratory-european bioinformatics institute
CNV: Copy number variation
GISA: Gestion integree de la sante animaux
EPIDEWILD-3i: Emergence of a pig disease in wild boars in a context of increasing interspecific interactions
GeT: Genomic and transcriptomic
CRCT: Cancer research center of toulouse
